# Survey of the general public's attitudes toward advance directives in Japan: How to respect patients' preferences

**DOI:** 10.1186/1472-6939-7-11

**Published:** 2006-10-18

**Authors:** Hiroaki Miyata, Hiromi Shiraishi, Ichiro Kai

**Affiliations:** 1 Department of Healthcare Quality Assessment, Graduate School of Medicine, University of Tokyo, Japan; 2 Toyo University, Faculty of Human Life Design, Japan; 3 Department of Social Gerontology, School of Health Sciences and Nursing, Graduate School of Medicine, University of Tokyo, Japan

## Abstract

**Background:**

Japanese people have become increasingly interested in the expression and enhancement of their individual autonomy in medical decisions made regarding medical treatment at and toward the end of life. However, while many Western countries have implemented legislation that deals with patient autonomy in the case of terminal illness, no such legislation exists in Japan. The rationale for this research is based on the need to investigate patient's preferences regarding treatment at the end of life in order to re-evaluate advance directives policy and practice.

**Methods:**

We conducted a cross-sectional survey with 418 members of the general middle-aged and senior adults (aged between 40 and 65) in Tokyo, Japan. Respondents were asked about their attitudes toward advance directives, and preferences toward treatment options.

**Results:**

Over 60% of respondents agreed that it is better to express their wishes regarding advance directives (treatment preferences in writing, appointment of proxy for care decision making, appointment of legal administrator of property, stating preferences regarding disposal of one's property and funeral arrangements) but less than 10% of them had already done so. About 60% of respondents in this study preferred to indicate treatment preferences in broad rather than concrete terms. Over 80% would like to decide treatment preferences in consultation with others (22.2% with their proxy, 11.0% with the doctor, and 47.8% with both their proxy and the doctor).

**Conclusion:**

This study revealed that many Japanese people indicate an interest in undertaking advance directives. This study found that there is a range of preferences regarding how advance directives are undertaken, thus it is important to recognize that any processes put into place should allow flexibility in order to best respect patients' wishes and autonomy.

## Background

In recent times, paternalistic decision making practices and policies regarding life prolonging treatments have widely been replaced by an emphasis on patient participation, respect for autonomy, and quality of life [1,2]. Research indicates that honoring the treatment preferences of terminally ill patients is critical for the provision of high quality care at the end of life [3-6]. Advance care planning including living wills and health care proxies have become established to facilitate ways for patients to specify the kind of medical treatment they desire in the case of incapacitation. The designation and presence of a health care proxy is significantly associated with higher patient satisfaction with comfort care plans[7]. Patients' preferences are most often elicited through advance directives, and discussions regarding advance directives could provide a framework for all treatment related decision-making between medical professionals and patients in addition to protecting patients in the event of decisional incapacity[8].

In the United States, all 50 states and the District of Columbia have passed legislation on advance directives[8]. In the United Kingdom, although the British Medical Association cautiously approved the introduction of advance directives in a statement in May 1992 [9], there is still no legislation which deals with patient autonomy in the case of terminal illness. This is similar to the situation in Japan where, although the Japan Medical Association has officially declared that a patient's advance request for death with dignity should be respected [10], advance directives have no legal standing.

While the general public in Japan have become increasingly interested in the expression and enhancement of their individual autonomy in medical decisions made at the end of life[11,12], one study has suggested there are several possible barriers in the appropriate use of advance directives[13]. In dealing with terminal patients, moreover, another study indicated that only half of Japanese doctors gave priority to their patients' wishes for medical care regardless of the patient's competency[14]. In the light of this, there is a need to investigate patient's preferences regarding treatment at the end of life in order to re-evaluate advance directives policy and practice.

When patients are deprived of decisional capacity due to physical, mental and cognitive deterioration, family or relatives are often asked to make judgments on behalf of patients regarding treatment choices. A number of studies have found, however, that both family members and care professional show substantial inaccuracy in attempting to predict patients' life-sustaining treatment preferences [15-17]. In order to facilitate the proxy decision making process, it is also useful to clarify what patients expect from surrogate decision makers with regards to end-of-life decision-making.

More than 103,000 people have joined the Japan Society for Dying with Dignity and have formulated a living will [18]. Though the advance directives format used by the Society gives no choice in terms of declaring wishes regarding treatment options, several researchers in the advanced directives field have pointed out that the desirability of making an advance directive depends on its outcome (e.g. life expectancy, chance of success) [19-21]. As both qualitative[22] and quantitative data [23,24] support the importance of outcomes in patients' treatment preferences, recent research indicates that advance directives should take into account patients' attitudes toward the burden of treatment, the possible outcomes, and their likelihood [25]. In order to examine the suitability of processes for advance directives from the patient's perspective in Japan, we conducted a population-based survey to clarify the general public's preferences to treatment with differing burden, outcomes and likelihood assuming that this group might best represent patients' attitudes.

## Methods

This study was a cross-sectional, stratified random sample survey of the general middle-aged and senior adults (aged between 40 and 65) in Tokyo, Japan. As people over 65 years old are epidemiologically more at risk of having dementia, we excluded them from the sample not only because it seemed harmful to ask them about dementia-related matters, but also because there was a possibility that their responses would be affected by any existing symptoms. Participants were selected from the list of eligible voters in A ward in Tokyo (which consists of 23 wards). This ward was selected as representative of Tokyo because various social indices such as the proportion of the elderly in the population, the average length of education, and the population growth rate were consistent with the Tokyo average[26]. A self-administered questionnaire was sent via mail to 688 residents in March 2004. Of these, 418 people responded (response rate 60.8 %; we made two times requests for participation by letter). The 12 page questionnaire was developed in consultation with 5 medical professionals and 30 lay people who give advice from the patient's perspective.

Respondents were asked about their preferences and attitudes toward advance directives by multiple answers. Questionnaire items include those relating to how they would like their treatment preferences to be made (verbally or in writing), the appointment of a proxy for health care decision-making, and the appointment of a legal executor for dealing with property, and statement of preferences regarding disposal of property and funeral arrangements. Respondents were asked whether they would like to express their preferences in advance toward each item, or whether they have already expressed their preferences.

Respondents were also asked about their preferences toward treatment preferences that were translated from American actual living will declarations17. The actual sentences are: 'If my condition is determined to be terminal and incurable, I do want life-sustaining procedures that serve only to prolong the process of my dying.', 'If I am in an irreversible or incurable persistent vegetative state, I do want cardiac resuscitation.', 'If I am in an irreversible or incurable persistent vegetative state, I do want artificial nutrition and hydration.', 'I want my life to be prolonged to the greatest extent possible.' The questionnaire asked respondents to what extent they wanted to express their preferences regarding treatment, how they would like to discuss treatment preferences with their proxy, and to what extent their proxy should respect the patient's treatment preferences.

The questionnaire presented a hypothetical scenario asking respondents about their preferences toward certain types of treatments in the event that they have limited capacity to make decisions due to illness. We created three types of scenarios with different diagnoses: dementia, irreversible coma, and temporary illness. One of the three scenarios was randomly assigned to each questionnaire so that each respondent answered questions regarding one situation only. The response rate toward each scenario was quite similar with 60.7% (142/235) responses to the dementia scenario, 59.5% (135/227) responses to the irreversible coma scenario, and 62.1% (141/227) responds to the temporary illness scenario. Questions were identical in all scenarios. The questionnaire began with the introduction: "If you acquired another illness while having moderate/severe dementia, would you choose to undergo the following medical treatments?" Respondents were then asked about their preferences toward a total of 8 active treatments (ATs) which have varying degrees of burden/chance of success/length of survival. As former research indicates that advance directives should take into account patients' attitudes toward the burden of treatment, the possible outcomes, and their likelihood25, we took these attributes into account when developing the AT options for the questionnaire. Five medical doctors and 30 lay people checked the validity, adequacy and understandability of the expressions used. No specific treatment was mentioned in the eight AT variations, but they were defined by the combination of the following characteristics: burden the AT imposes is low or high, the chance of success is good or poor, and possible outcome in terms of the length of survival is about 6 months when not treated as opposed to over 2 years when treated in one version, less than 1 month when not treated as opposed to about 6 months when treated in another version. Examples of low-burden approaches were described as therapies such as oral administration of medication and intravenous antibiotics. Those of high-burden approaches were described as surgery and medication with possibly severe side effects.

Respondents were also asked about their preferences toward 8 life-sustaining treatments (LSTs): 4 types of LST with two different survival periods. The types of LST included were cardio-pulmonary resuscitation, artificial ventilation, dialysis, and artificial nutrition. Each treatment prolongs the length of survival to over 2 years in one version, and to about 6 months in another. The scenarios stated that without these LSTs the patient would die within a short time. The questions were intended to investigate whether respondents' preferences toward each type of the LSTs would change if the length of survival is different. As for the ATs, in which no treatment was specified, respondents had to give their preferences in relation to burden, chance of success and survival period, but for the LSTs, the respondents had to give preferences regarding the specific treatments and the length of survival.

The questionnaire also included the General Health Questionnaire 12 item Japanese version (GHQ-12), which assesses predisposition to non-psychotic psychiatric illness27,28. The Japanese version of GHQ-12 is standardized and widely used29,30. In the present sample, the GHQ for Cronbach's α = 0.84.

We described the distributions of the study population, their attitudes regarding advance directives and treatment preferences. The Kruskal-Wallis test, χ^2 ^test and Fisher exact test were used to determine the differences in respondents' characteristics among the three scenario groups. Age was divided into one group consisting of respondents aged 53 years (mean age in this study) and another group aged 53 years and under. Scores regarding GHQ were also analyzed according to 2 groups consisting of the group of respondents who scored above 25 (the mean total score of respondents in this study) and the group of respondents who scored 25 and under. To examine the relationship between respondents' characteristics and their preferences or attitudes toward treatments, we used the total score of the LSTs and the ATs and compared them with patients' characteristics (Range 0–8; for each item, negative attitude = 0, positive attitude = 1). Mann-Whitney's U-test was used to examine this relationship for each scenario group. For each scenario group factor analysis using a promax rotation was used to identify the underlying dimensions of the 16 treatment items: 8 variations of ATs and 4 types of LST with two different survival periods. χ^2 ^test was used to determine the differences in treatment preferences among the three scenario groups. All reported p values are two-sided. Statistical analyses were conducted using SPSS Version 13.0J.

## Results

The characteristics of respondents are shown in Table 1. A total of 418 people responded with a mean age of 52.8 years ± 7.4 years. There were no significant differences in the characteristics of respondents between the three scenario groups. Respondents' attitudes toward advance directives are shown in Table 2. Over 70% of respondents would like to express treatment preferences orally and would like to appoint a proxy for making care related decisions.

**Table 1:** Characteristics of respondents (N = 418)

	**Mean**	**SD**
Age (yrs)	52.8	± 7.4
GHQ (total score)	24.7	± 5.0

	**N**	**%**

Sex (female)	241	(57.7)
College Graduates	114	(27.3)
Living Alone	51	(12.2)
Married	319	(76.3)
Living with Adult Child	153	(36.6)
Living with Infant Child	127	(30.4)
No Religion	324	(77.5)

**Table 2:** Patient's attitudes toward advance directives (N = 418) ***MA***

Items for determination	would like to express his/her preferences		have already expressed his/her preferences	
		
	N	%	N	%
Treatment preferences (orally)	308	73.7	85	20.3
Treatment preferences (in writing)	251	60.0	11	2.6
Appointing proxy of care decision	296	70.8	17	4.1
Appointing legal administrator of property	253	60.5	12	2.9
Disposal of one's property	262	62.7	10	2.4
Funeral arrangements	255	61.0	38	9.1

Regarding preferences about medical treatments based on the American format for Advance Directives, 72.7% of respondents did not want life-sustaining procedures that served only to prolong the process of dying if their condition is determined to be terminal and incurable, 81.6 % did not want cardiac resuscitation if they are in an irreversible or incurable persistent vegetative state, 78.5% of them did not want artificial nutrition and hydration if they are in an irreversible or incurable persistent vegetative state, and 6.9% of respondents wanted their life to be prolonged to the greatest extent possible.

Regarding the process for deciding treatment preferences, 73 (17.5%) respondents wanted to decide 'by oneself', 93 (22.2%) wanted to decide with their proxy (family or friend), 46 (11.0%) wanted to decide with the doctor, and 200 (47.8%) wanted to decide with both their proxy and the doctor. The degree of detail that respondents wished preferences to be recorded in their advance plan also showed strong variation with 56 (13.4%) respondents stating that they 'definitely wanted' to describe their treatment preferences in concrete terms, 104 (24.9%) respondents 'preferred' to describe their treatment preferences in concrete terms, while 141 (33.7%) 'definitely wanted' to indicate treatment preferences in broad terms, and 109 (26.1%) 'preferred' to indicate treatment preferences in broad terms".

Regarding how often and when patients would like to discuss their treatment preferences with their proxy, 106 (25.4%) chose "regularly" and 214 (51.2%) chose "when clinical conditions require", while 60 (14.4%) chose "once", 20 (4.8%) chose "in absolute necessity" and 10 (2.4%) said "never". Concerning to what extent their proxy should respect their treatment preferences, 21 (5.0%) said their preferences should be ''strictly observed" and 243 (58.1%) said "as much as possible," while 137 (32.8%) respondents said "only as a reference", 11 (2.6%) said "they would not care even if their preferences are not observed".

Responses to preferences in the event of dementia found that respondents who were married (p < 0.05) and who lived with an infant child (p < 0.01) were more likely to have positive attitudes toward ATs. Respondents' characteristics did not affect responses to treatment preferences in the case of the irreversible coma scenario. As for temporary illness, respondents aged over 53 (p < 0.01) and who are female (p < 0.05), who lived with an adult child (p < 0.01), not living with an infant child (p < 0.01) were more likely to have negative attitudes toward ATs. Respondents who lived with an adult child (p < 0.05), who did not live with a infant child (p < 0.05) also have negative attitudes toward LSTs in the temporary illness scenario.

The distributions of ATs preferences based on three hypothetical scenarios were shown in Table 3. Among the eight ATs variations, the three scenario groups showed no significant differences in responses to the following four types of ATs: 1) a treatment that imposes high burden, has poor chance of success and provides a 6-month life expectancy when successfully treated and a 1 month life expectancy when not treated, 2) a treatment that imposes high burden, has poor chance of success and provides over a two-year life expectancy when successfully treated and a 6-month life expectancy when not treated, 3) a treatment that imposes high burden, good chance of success and provides a 6-month life expectancy when successfully treated and a 1-month life expectancy when not treated, 4) a treatment that imposes low burden, has poor chance of success and provides a 6-month life expectancy when successfully treated and a 1-month life expectancy when not treated. The distributions of LSTs preferences based on three hypothetical scenarios were shown in Table 4. Among the eight LSTs variations, the three scenario groups showed significant differences in responses to preferences for the different types of treatments.

**Table 3:** Distribution of patients who want treatment (N = 418)

Burden	high	high	high	high	Low	low	low	low
Chance of success in the treatment	poor	poor	good	good	Poor	poor	good	good
Life expectancy (success/failure)	6 mo./1 mo.	+ 2 yrs/6 mo.	6 mo./1 mo.	+2 yr/6 mo.	6 mo./1 mo.	+2 yr/6 mo.	6 mo./1 mo.	+ 2 yr/6 mo.
	**% (distribution of patients who want treatment)**							
**Dementia**	4.4 % (6)	4.4 % (6)	17.6 % (24)	29.9 % (41)	27.7 % (38)	31.4 % (43)	65.7 % (90)	76.1 % (105)
**Irreversible Coma**	6.1 % (8)	5.3 % (7)	18.0 % (24)	16.5 % (22)	24.2 % (32)	26.3 % (35)	50.4 % (67)	64.4 % (87)
**Temporary Illness**	3.0 % (4)	8.3 % (11)	18.9 % (25)	38.3 % (51)	29.3 % (39)	45.1 % (60)	74.5 % (102)	94.9 % (131)
	N.S.	N.S.	N.S.	**p < .001**	N.S.	**P < .01**	**p < .001**	**p < .001**
	χ^2 ^= 1.44	χ^2 ^= 2.02	χ^2 ^= 0.79	χ^2 ^= 15.9	χ^2 ^= 0.91	χ^2 ^= 11.2	χ^2 ^= 17.3	χ^2 ^= 38.5

**Table 4:** Distribution of patients who want life-sustaining treatment (N = 418)

	Artifical Ventilator	Artifical Ventilator	CPR	CPR	Dialysis	Dialysis	Artificial Nutrition	Artificial Nutrition
Life expectancy	6 mo.	+ 2 yr	6 mo.	+ 2 yr	6 mo.	+ 2 yr	6 mo.	+ 2 yr
	**% (distribution of patients who want treatment)**							
**Dementia**	23.2 % (32)	29.0 % (40)	27.5 % (38)	36.2 % (50)	25.4 % (35)	33.3 % (46)	26.8 % (37)	33.3 % (46)
**Irreversible Coma**	20.9 % (28)	24.6 % (33)	23.9 % (32)	29.9 % (40)	23.1 % (31)	27.6 % (37)	29.9 % (40)	32.8 % (44)
**Temporary Illness**	44.4 % (60)	59.3 % (80)	52.2 % (71)	64.7 % (88)	48.9 % (66)	67.4 % (91)	46.3 % (63)	61.0 % (83)
	**p < .001**	**p < .001**	**p < .001**	**p < .001**	**p < .001**	**p < .001**	**p < .01**	**p < .001**
	χ^2 ^= 21.9	χ^2 ^= 41.0	χ^2 ^= 28.5	χ^2 ^= 38.0	χ^2 ^= 25.1	χ^2 ^= 51.1	χ^2 ^= 13.3	χ^2 ^= 29.0

Factor analysis was conducted on each scenario group (see Table 5, 6, 7). In the dementia scenario (Table 5) and temporary illness scenario (Table 7), Factor 1 items were composed of LSTs and Factor 2 items were composed of 4 types of ATs all of which imposed high burden. In the irreversible coma scenario (Table 6), Factor 1 items were composed of 4 types of LSTs all of which provide over 2 years life expectancy and Factor 2 items were composed of 4 types of LSTs all of which provide about 6 months life expectancy. Eight type of LSTs accounted for the nearly half of the total variance in the analyses conducted on each scenario (Dementia 45.6%, Irreversible Coma 57.4%, Temporary illness 41.6%).

**Table 5:** Factor analysis toward 16 treatment variations in dementia scenario. (N = 142)

Items	Factor 1	Factor 2	Factor 3	Factor 4
*Variance*	*45.6%*	*14.8%*	*9.7%*	*6.9%*
Low burden, Good chance, 2 years	.372	.199	**.906**	.213
High burden, Good chance, 2 years	**.518**	**.662**	**.596**	
Low burden, Poor chance, 2 years	.201	.319	.330	**.903**
High burden, Poor chance, 2 years	.207	**.869**	.130	.331
Low burden, Good chance, 6 months	.458	.249	**.894**	.303
High burden, Good chance, 6 months	**.560**	**.778**	.471	
Low burden, Poor chance, 6 months	.170	.322	.277	**.926**
High burden, Poor chance, 6 months	.207	**.869**	.130	.331
CPR, 2 years	**.815**	.262	.446	.113
Artificial Ventilator, 2 years	**.866**	.319	.468	.203
Dialysis, 2 years	**.822**	.303	.502	.115
Artificial Nutrition, 2 years	**.826**	.340	.424	
CPR, 6 months	**.835**	.322	.409	
Artificial Ventilator, 6 months	**.884**	.347	.386	.116
Dialysis, 6 months	**.886**	.356	.419	
Artificial Nutrition, 6 months	**.872**	.361	.403	

* All loadings greater than 0.50 are in bold type

**Table 6:** Factor analysis toward 16 treatment variations in irreversible coma scenario. (N = 135)

Items	Factor 1	Factor 2	Factor 3	Factor 4
*Variance*	*46.0%*	*11.4%*	*8.4%*	*6.7%*
Low burden, Good chance, 2 years	.400	.468	.**780**	.182
High burden, Good chance, 2 years	.262	.388	.452	**.783**
Low burden, Poor chance, 2 years	.326	.148	**.755**	.495
High burden, Poor chance, 2 years	**.505**	.126	.266	**.780**
Low burden, Good chance, 6 months	.258	**.513**	**.862**	.282
High burden, Good chance, 6 months	.212	**.630**	.456	**.698**
Low burden, Poor chance, 6 months	.361	.185	**.758**	**.535**
High burden, Poor chance, 6 months	.381	.408	.313	**.806**
CPR, 2 years	**.905**	.481	.321	.373
Artificial Ventilator, 2 years	**.906**	.497	.374	.421
Dialysis, 2 years	**.732**	**.534**	.524	.290
Artificial Nutrition, 2 years	**.754**	**.617**	.438	.295
CPR, 6 months	**.691**	**.784**	.259	.412
Artificial Ventilator, 6 months	**.627**	**.778**	.310	.411
Dialysis, 6 months	.470	**.879**	.387	.267
Artificial Nutrition, 6 months	**.546**	**.827**	.400	.339

* All loadings greater than 0.50 are in bold type

**Table 7:** Factor analysis toward 16 treatment variations in temporary illness scenario. (N = 141)

Items	Factor 1	Factor 2	Factor 3	Factor 4
*Variance*	*41.6%*	*11.8%*	*8.1%*	*7.0%*
Low burden, Good chance, 2 years	.261	.120		**.735**
High burden, Good chance, 2 years	**.500**	**.744**		.381
Low burden, Poor chance, 2 years	.297	.333	**.832**	.186
High burden, Poor chance, 2 years	.132	**.806**	.287	
Low burden, Good chance, 6 months	.366	.113	.303	**.808**
High burden, Good chance, 6 months	.397	**.681**		.485
Low burden, Poor chance, 6 months	.310	.230	**.885**	.231
High burden, Poor chance, 6 months	.174	**.683**	.362	
CPR, 2 years	**.840**	.298	.240	.382
Artificial Ventilator, 2 years	**.830**	.256	.218	.334
Dialysis, 2 years	**.802**	.274	.177	.233
Artificial Nutrition, 2 years	**.781**	.382		.273
CPR, 6 months	**.867**	.253	.297	.489
Artificial Ventilator, 6 months	**.859**	.227	.310	.459
Dialysis, 6 months	**.824**	.267	.224	.435
Artificial Nutrition, 6 months	**.842**	.347	.188	.402

* All loadings greater than 0.50 are in bold type

## Discussion

This study revealed that many middle-aged and senior people in Tokyo indicate an interest in undertaking advance directives. Nearly three-quarters (73.7%) of respondents in this study said that they would like to verbally express their treatment preferences in advance. This result is reflected in other research conducted in Japan which found that 80.5% of a general public sample strongly/moderately agreed with advance directives [12]. However the fact that only 20.3% of the people in our study had already undertaken advance directives indicates that despite a interest, barriers exist to carrying this out (Table 2). While there is strong recognition of the benefits of undertaking advance directives with over 60% of respondents agreed that it is preferable to express their wishes regarding the appointment of a proxy for care decisions, appointment of a legal administrator of property, stating preferences regarding one's property and funeral arrangements, less than 10% of them had already done so (Table 2). The existence of a number of barriers to undertaking advance directives has been identified in the literature. Research indicates that Japanese people have difficulty in actually expressing their preferences regarding advance directives even in the case that they are willing do so[32] Furthermore, dementia has been identified as contributing to difficulties in encouraging advance directives policies. Our former study showed that of the nursing homes in Japan that confirm their residents' preferences regarding advance directives, for the most part, this is only undertaken some time after patients have been admitted [31]. However, this may be inappropriately late given that more than a majority of residents have difficulties in daily life due to dementia. While it is clear that it is highly desirable for patients to undertake advance directives before dementia has reached advanced stages, in many cases, treatment preference made might not be respected because of patients' difficulties in expressing preferences and nursing home attitudes that do not validate advance directives in a positive way. In order to ensure that the treatment preferences of the general public in Japan are recognized and met, some support for individuals as well as nursing homes and medical professionals is necessary.

About 60% of respondents in this study preferred to indicate treatment preferences in broad rather than concrete terms. Over 80% would like to decide treatment preferences in consultation with others (22.2% with their proxy, 11.0% with the doctor, and 47.8% with both their proxy and the doctor) and over 75% of respondents would like the opportunity to discuss their treatment preferences with their proxy more than once (51.2% when clinical conditions require, 25.4% regularly). Respondents responses reflected a range of preferences regarding how advance directives are undertaken, thus it is important to recognize that any processes put into place should allow flexibility in order to best respect patients' wishes and autonomy. In the case of shared treatment decision-making, further investigation is required to examine what processes would be appropriate in order to respond to patients and their surrogate decision makers' desires.

Even in the case of advance directives that does not specify detail, patient wishes do need to distinguish between preferences that arise in the cases of different kinds of complications that occur toward the end of life including irreversible coma, dementia and temporary illness. Respondents' preferences varied amongst the scenarios for the four active treatments and all the life-sustaining treatments. Moreover results of factor analysis for three of the scenarios are slightly different with patient preferences ranged from high rates of desire to receive treatments in the event of temporary illness to high rates of treatment rejection in the case of irreversible coma. Similar results have also been reported elsewhere [33,34]. In research conducted in Japan on advance directives making in the event of terminal illness, Asai [19] suggested that Japanese terminally patients, despite having competency to do so, might not actually be able to make their advance directives concrete enough to guide physician's decisions. Given these findings, it might be need to consider distinguishing diagnoses for which a Japanese advance directive format applies or does not apply.

This research found that the burden of treatment and the likelihood of outcome all influenced treatment preferences, consistent with results found in another study [25]. The results of the factor analysis on preference suggest that patient' preferences toward LSTs and burden of treatments should always be confirmed when asking about treatment preferences in end-of-life situation. However LSTs accounted for the nearly half of the total variance in the analyses conducted on each scenario. As four types of high burden ATs were Factor 2 in both the dementia and temporary illness scenarios and 4 types of low burden ATs were Factor 3 in the irreversible coma scenario, the second biggest factor affecting preferences might be the perceived level of burden of ATs. If patients would like to indicate treatment preferences in a broad sense rather than concrete terms, it might be possible to do so by putting all LSTs and high burden treatments in one category, without specifying treatment. Further studies will be required to confirm whether this simplification is appropriate or not.

This research's results suggest that type of diagnosis given to the general public members in this sample and their characteristics have little effect when they have only severe treatment outcome. Three of the scenario groups showed no significant differences in preferences to four types of active treatments, of which at least two had negative attributes of burden, chances of success and life expectancy. On the other hand respondents' characteristics did not affect treatment preferences in the irreversible coma scenario, while many characteristics significantly affected preferences in the other two scenarios. Many respondents refused high burden, poor likelihood treatments even in temporary illness scenario which is relatively healthy state. As temporary illness is defined "decisional incapacity state cause by such as mental disorders, and people would soon recover capacity", respondents might consider their choice only for incapacity period.

As this study is a cross sectional survey in Japan, these results may not be simply applicable to other country. Though the desire for group decision making might be different in Japan in comparison to Western countries, a former study suggests that Japanese patients' preference for disclosure, willingness to forgo care, and views regarding advance directives are shifting toward those found in the West [35]. As respondents' preferences regarding life-sustaining treatment in this study (23%–36% wanting LSTs in the case of dementia;21%–33% wanting LSTs in the cse of Coma) are similar to those found in the United States (23% to 42% wanting LSTs in the case of dementia; about 14–29) wanting LSTs in the case of Irreversible coma 17, 23, our previous study shows a similar tendency between the two countries regarding patients' preferences and cancer disclosure[36]. Despite differences between Japanese and western and US medical institutional policies and doctors' attitudes regarding advance directives [37], it is suggested that there is little difference in patients' side preferences regarding treatment preferences.

We would like to make a couple of comments on the representative-ness of this survey's sample. The response rate for this study was generally good for a general population survey, though slight lower that of a similar Japanese study which found that 80.5% of respondent strongly/moderately agreed with advance directives12. We therefore believe that the response rate did not significantly affect the overall results obtained in our study. However this study's sample was limited to residents who reside in Tokyo aged between 40 and 65. As the Tokyo is the most condensed and diverse metropolitan area in Japan, further research is needed in order to collect and comparing our results with data collected in rural areas and smaller towns and cities. It is also required to add younger (under the age of 40) and older people (over the age of 65) in further research to consider generalization of these findings.

In order to respect Japanese patients' preferences, it might be important to implement flexible processes and practices that are able to accommodate patients' preferences as well as nursing homes and medical professionals needs regarding advance directives and surrogate decision making. Specific to the Japanese context, the results of this survey indicate that advance directives might need to differentiate processes according to diagnoses including LSTs and high burden ATs treatment items.

## Conclusion

This study revealed that many Japanese people indicate an interest in undertaking advance directives.  This study found that there is a range of preferences regarding how advance directives are undertaken, thus it is important to recognize that any processes put into place should allow flexibility in order to best respect patients’ wishes and autonomy.

## Authors' contributions

HM planned and conducted the survey, carried out the analysis, and wrote this paper. IK and HS assisted with the development, analysis and writing of this paper. All authors read and approved the final manuscript.

## Pre-publication history

The pre-publication history for this paper can be accessed here:


